# Gastroenteritis and Miliary Lung Opacities: An Interesting Combination of Findings

**DOI:** 10.7759/cureus.8848

**Published:** 2020-06-26

**Authors:** Umer Farooq, Muhammad Anwar, Daniel Alcantar, Mukarram Amine, Daniel Colon Hidalgo

**Affiliations:** 1 Internal Medicine, Loyola Medicine/MacNeal Hospital, Berwyn, USA; 2 Internal Medicine, Yale Waterbury, Waterbury, USA; 3 Internal Medicine, MacNeal Hospital, Berwyn, USA

**Keywords:** e-cigarette and vaping product use associated lung injury (evali), silent lung injury, gastrointestinal symptoms

## Abstract

A 19-year-old man with a one-year history of vaping with multiple emergency room visits for acute gastroenteritis like symptoms was noted to have asymptomatic hypoxia with a PaO2 of 65 mmHg. Computed tomography revealed bilateral nodular lung infiltrates. History was negative for travel, allergies, or animal exposure. An infectious work-up was negative for bacterial, viral, or fungal infections, including bronchoalveolar lavage sample cultures. He did not show improvement upon initial empiric antibacterial and antifungal treatment. His hypoxia improved with systemic steroids. E-cigarette-associated lung injury (EVALI) is a diagnosis of exclusion, and unfortunately, may produce prolonged gastrointestinal symptoms with clinically silent but severe lung injury.

## Introduction

E-cigarette or vaping product use associated lung injury (EVALI) has had a rising incidence since the first case was reported in June 2019. A study in the same year reported over five million school going students used e-cigarettes in the past 30 days [1]. Sixty-eight deaths have been reported by the Centers for Disease Control and Prevention (CDC) caused by EVALI as of February 2020 [2]. We report a similar case which presented with minimal pulmonary symptoms.

## Case presentation

**Figure 1:**
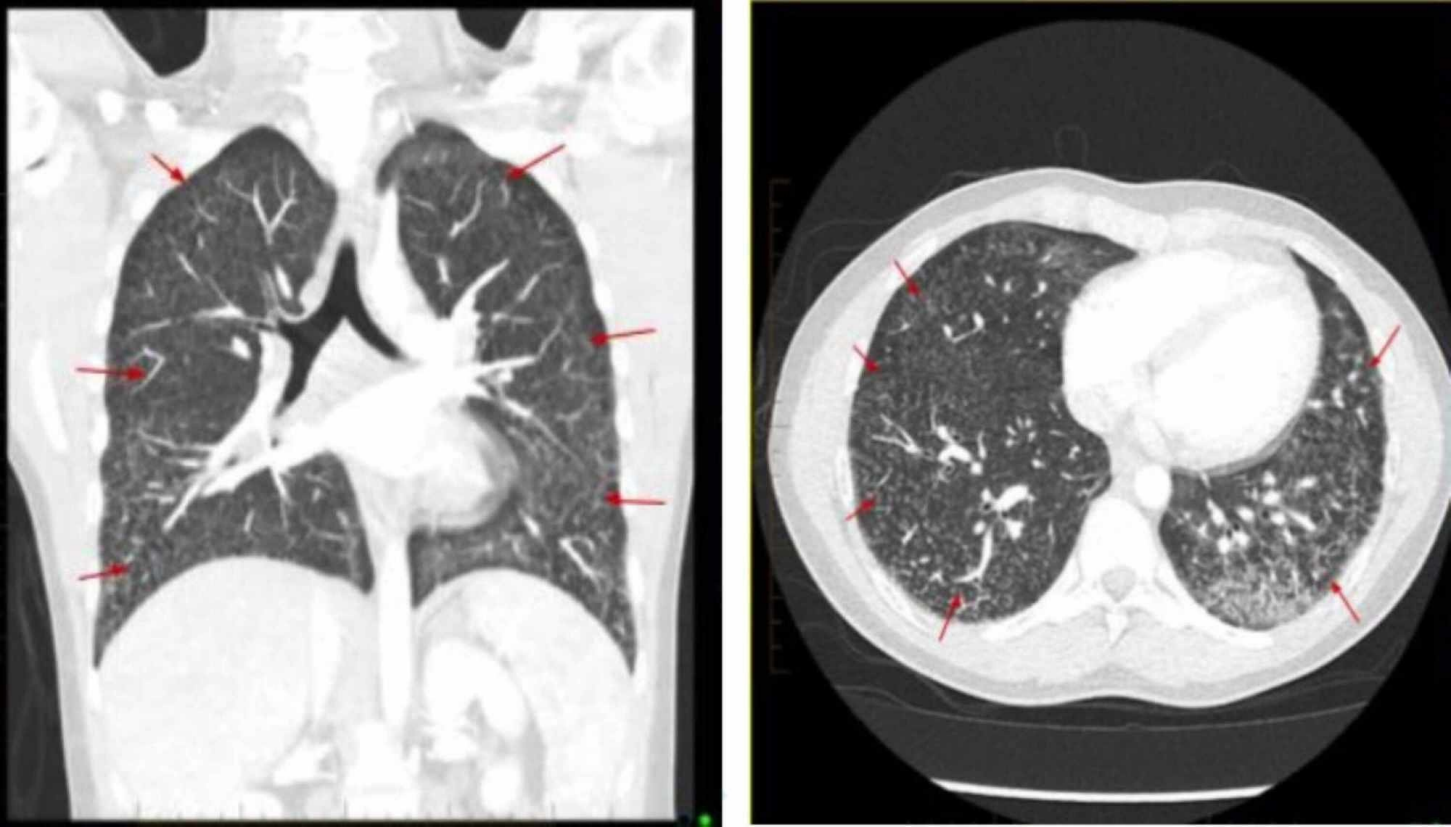
CT chest

A 19-year-old man with a one-year history of intermittent use of nicotine-containing vaping products was being evaluated for acute gastroenteritis. He had multiple ED visits spanning over four months due to recurrent diffuse abdominal pain associated with occasional nausea, vomiting, and diarrhea, but laboratory and imaging work-up did not reveal any intra-abdominal source of pathology. In the hospital, he was noted to have asymptomatic hypoxia with a PaO2 of 65 mmHg. Physical examination did not reveal any pulmonary abnormality. A chest x-ray followed by a chest CT scan was ordered, which revealed a bilateral diffuse nodular pattern of infiltrates similar to miliary tuberculosis (Figure 1).

The differential diagnoses were very broad at this point, including Streptococcus pneumoniae, Legionella pneumoniae, Mycoplasma pneumoniae, Mycobacterial infections, Chlamydophila psittaci, Rickettsia, HIV, Cryptococcus, Histoplasmosis, Blastomycosis with EVALI being the top differential. An infectious work-up, including Streptococcus and Legionella urinary antigens, HIV screening, and Quantiferon testing, was negative. He underwent bronchoscopy with bronchoalveolar lavage (BAL), and bacterial and fungal cultures were negative. BAL specimen testing was also negative for viral, bacterial, or mycobacterial organisms. Urine antigen testing for Blastomyces and Histoplasma was negative, as well as Chlamydophila psittaci and Rickettsia antibody panels were negative. The patient was initially empirically treated for bacterial and fungal infection but showed minimal improvement. Systemic steroids were eventually added with the guidance from the Pulmonary and Infectious Disease services, after which the patient’s oxygen saturation started to improve. The patient received methylprednisolone 60 mg IV on day 1, followed by five days of prednisone 40 mg orally. Empiric antibiotic and antifungal therapy was discontinued, given his negative microbiologic work-up. His hypoxia continued to improve with systemic steroids, which he continued upon discharge on a slow tapering regimen over two weeks. Upon follow-up, he continued to remain asymptomatic while abstinent from the use of e-cigarettes.

## Discussion

Even though gastrointestinal symptoms account for the second most commonly found symptoms in patients with EVALI, it is rare for patients to present with such symptoms alone for a considerable time without pulmonary complaints. Our patient had multiple emergency visits with the chief complaint of abdominal pain but demonstrated stable vitals and work-up, including contrast-enhanced CT, and urine drug screen was always negative. BAL fluid samples from EVALI patients have found tetrahydrocannabinol (THC) and vitamin E acetate, along with the combustion of cannabinoid or coconut oil playing a role in the pathogenesis of EVALI [3]. Our patient responded dramatically to the first dose of IV steroids with improvement in hypoxia, so BAL toxicology workup was deferred. Preliminary studies have shown that nicotine is found in BAL samples of only 64% of EVALI patients who used nicotine vapes, and nicotine can be present in BAL samples of asymptomatic people who smoked regular cigarettes as well [4].

This patient’s course was overlapping with that of sarcoidosis, but the absence of infectious respiratory symptoms, greater mid and lower lobe involvement on imaging compared to upper lobe, absence of extra-pulmonary finding, dramatic response to steroids (two weeks for this patient with EVALI vs. four to six weeks for sarcoidosis), normal pulmonary function testing (PFT) (normal PFTs at follow-up for this patient vs. 65% of patients with pulmonary sarcoidosis have abnormal PFTs), and temporal relationship of symptoms with vaping favored EVALI over sarcoidosis.

EVALI is a diagnosis of exclusion, and unfortunately, as highlighted in this case, may produce clinically silent but severe lung injury. Clarifying the use of vaping while taking social history is critical due to the lack of emphasis in media on health hazards associated with vaping. Patients may not volunteer to provide the information which could delay diagnostic work-up. Timely diagnosis and appropriate management can reduce the need for ICU admission, intubation, and overall cost of care. Empiric antibiotics should be initiated in parallel with obtaining microbiological workup and discussion with specialists on the proper timing of steroid therapy. There is a wide range of steroid dosing in current studies. Physicians should always consider treating with the lowest dose possible, depending on the patient’s clinical response to limit medication adverse effects. Routine screening chest X-ray in patients who use E-cigarettes and other vaping related products is a potential area to study given that EVALI can manifest as a wide range of symptoms from clinically silent to acute hypoxic respiratory failure requiring mechanical ventilation.

## Conclusions

In vaping patients who present with non-specific symptoms, we should maintain a high suspicion for EVALI. A thorough assessment can prompt early diagnosis and intervention. A patient’s pulmonary symptoms may not correlate to radiological lung findings. In the limited published literature available on EVALI, empiric antibiotics coupled with appropriate use of systemic glucocorticoids have shown to result in favorable outcomes.
